# Histopathology images-based deep learning prediction of prognosis and therapeutic response in small cell lung cancer

**DOI:** 10.1038/s41746-024-01003-0

**Published:** 2024-01-18

**Authors:** Yibo Zhang, Zijian Yang, Ruanqi Chen, Yanli Zhu, Li Liu, Jiyan Dong, Zicheng Zhang, Xujie Sun, Jianming Ying, Dongmei Lin, Lin Yang, Meng Zhou

**Affiliations:** 1 Department of Pathology, National Cancer Center/National Clinical Research Center for Cancer/Cancer Hospital, Chinese Academy of Medical Sciences and Peking Union Medical College, Beijing, 100021 P. R. China; 2 School of Biomedical Engineering, Wenzhou Medical University, Wenzhou, 325027 P. R. China; 3 Key Laboratory of Carcinogenesis and Translational Research (Ministry of Education), Department of Pathology, Peking University Cancer Hospital and Institute, Beijing, 100142 P. R. China

**Keywords:** Prognostic markers, Small-cell lung cancer, Image processing

## Abstract

Small cell lung cancer (SCLC) is a highly aggressive subtype of lung cancer characterized by rapid tumor growth and early metastasis. Accurate prediction of prognosis and therapeutic response is crucial for optimizing treatment strategies and improving patient outcomes. In this study, we conducted a deep-learning analysis of Hematoxylin and Eosin (H&E) stained histopathological images using contrastive clustering and identified 50 intricate histomorphological phenotype clusters (HPCs) as pathomic features. We identified two of 50 HPCs with significant prognostic value and then integrated them into a pathomics signature (PathoSig) using the Cox regression model. PathoSig showed significant risk stratification for overall survival and disease-free survival and successfully identified patients who may benefit from postoperative or preoperative chemoradiotherapy. The predictive power of PathoSig was validated in independent multicenter cohorts. Furthermore, PathoSig can provide comprehensive prognostic information beyond the current TNM staging system and molecular subtyping. Overall, our study highlights the significant potential of utilizing histopathology images-based deep learning in improving prognostic predictions and evaluating therapeutic response in SCLC. PathoSig represents an effective tool that aids clinicians in making informed decisions and selecting personalized treatment strategies for SCLC patients.

## Introduction

Lung cancer represents the most commonly diagnosed malignant tumor worldwide^1^. Among the different subtypes, small cell lung cancer (SCLC) accounts for ~15–20% of all lung cancer cases, and is characterized by its highly invasive neuroendocrine nature, rapid growth, early metastasis, frequent recurrence, and strong resistance to drugs^2,3^. Despite advancements in therapy, the prognosis for SCLC remains grim, with a dismal five-year survival rate of less than 10%^3^, highlighting the urgent need for improved prognostic tools and personalized treatment strategies. The current clinical and pathological features used for prognostic assessment and treatment decision-making in SCLC have certain limitations, especially in predicting individual patient responses and survival outcomes. Several efforts have been made to uncover the complex heterogeneity of the disease, including investigations into neuroendocrine differentiation, transcriptionally defined subtypes and tumor microenvironment features^4–7^. Although this knowledge has greatly improved our understanding of the molecular mechanisms underlying SCLC heterogeneity and provided prognostic and theragnostic implications to some extent, their heterogeneity application in clinical trials and routine patient care is limited by several challenges, including the quantity and quality of the samples, trans-platform reproducibility, expensive and time-consuming.

Histopathological examination of tissue slides is pivotal in cancer diagnosis and treatment planning. Hematoxylin and Eosin (H&E) staining, a widely adopted technique in pathology laboratories, provides high-resolution images that capture essential morphological features of tumor tissues. However, the manual microscopic examination of H&E-stained slides heavily relies on the expertize of pathologists, making it labor-intensive and experience-dependent. To address these limitations, there is a growing interest in leveraging advanced technologies, such as deep learning and computer image processing, to extract valuable biological information from pathological slides beyond routine diagnostics. Specifically, recent advancements made in deep learning for computational pathology have enabled the use of H&E-stained slides for automated cancer detection and differential diagnosis^8^, quantification of morphologic phenotypes, and prediction of patient survival stratification in various cancers^9,10^. However, the application of artificial intelligence (AI) algorithms in the field of SCLC digital pathology remains relatively limited and warrants further exploration.

In this study, we propose unsupervised deep learning with contrastive clustering computational framework (DL-CC) to extract and analyze histomorphological features from H&E-stained histopathological images, and develop a pathomics signature (PathoSig). The extensive validation experiments in multicenter retrospective datasets demonstrated the robustness and generalizability of PathoSig in predicting prognosis and assessing the clinical benefits associated with chemoradiotherapy in patients with SCLC.

## Results

### Patient characteristics and study design

The baseline characteristics of the 380 SCLC patients are summarized in Table 1. The PUCH cohort comprised 94 cases of pure SCLC (P-SCLC), while the CHCAMS cohort included 240 P-SCLC cases and 46 combined SCLC (C-SCLC) cases, such as SCLC combined with squamous cell carcinoma (*n* = 19, 41.3%), adenocarcinoma (*n* = 18, 39.1%), large cell carcinoma (*n* = 4, 8.7%), large cell neuroendocrine carcinoma (LCNEC, *n* = 2, 4.3%), carcinoid tumor (*n* = 1, 2.1%), carcinoid tumor and LCNEC (*n* = 1, 2.1%) and adenosquamous carcinoma (*n* = 1, 2.1%). Male predominance is observed across all cohorts (70% and 76.9% for P-SCLC and C-SCLC in the CHCAMS cohort and 71.28% for the PUCH cohort). The median (range) ages are 56.5 (19–82), 60 (39–76) and 59.5 (33–82) years, and median follow-up durations are 4.00, 4.69, and 3.33 years, and recurrence rates are 49.17%, 50% and 69.15% for CHCAMS-P-SCLC, CHCAMS-C-SCLC, and PUCH cohorts, respectively. In all cohorts, 141 (58.72%), 24 (52.17%) and 72 (76.60%) cases were in stage I–II, while 99 (41.25%), 22 (47.83%) and 22 (23.40%) cases were in stage III-IV, with lymphatic metastasis observed in 137 (57.08%), 30 (65.22%) and 37 (39.36%) cases across all cohorts.

**Table 1:** Clinical characteristics of SCLC patients used in multicenter study.

Clinical characteristics	CHCAMS cohort		PUCH cohort
	P-SCLC (*n* = 240)	C-SCLC (*n* = 46)	P-SCLC (*n* = 94)
Age, median (range)	56.5 (19–82)	60 (39–76)	59.5 (33–82)
Sex, *n* (%)			
Male	168 (70.00)	35 (76.09)	67 (71.28)
Female	72 (30.00)	11 (23.91)	27 (28.72)
Smoking, *n* (%)			
Yes	153 (63.75)	36 (78.26)	72 (76.60)
No	87 (36.25)	10 (21.74)	22 (23.40)
AJCC/UICC, *n* (%)			
I	76 (31.67)	9 (19.56)	47 (50.00)
II	65 (27.08)	15 (32.61)	25 (26.60)
III	93 (38.75)	20 (43.48)	22 (23.40)
IV	6 (2.50)	2 (4.35)	0 (0.00)
Treatment modes, *n* (%)			
Surgery alone	8 (3.33)	0 (0)	22 (23.40)
Postoperative chemoradiotherapy	183 (76.25)	41 (89.13)	51 (54.26)
Preoperative chemoradiotherapy	23 (9.58)	2 (4.35)	2 (2.13)
Preoperative and postoperative chemoradiotherapy	0 (0.00)	0 (0.00)	11 (11.70)
Unknown	26 (10.84)	3 (6.52)	8 (8.51)
Follow-up (years), median (range)	4.00 (0.00–13.83)	4.69 (0.33–14.08)	3.33 (0.25–9.17)
Recurrence, *n* (%)			
Yes	118 (49.17)	23 (50)	65 (69.15)
No	122 (50.83)	23 (50)	29 (30.85)
Lymphatic metastasis, *n* (%)			
Yes	137 (57.08)	30 (65.22)	37 (39.36)
No	103 (42.92)	16 (34.78)	57 (60.64)
Death event, *n* (%)			
Yes	92 (38.33)	18 (39.13)	59 (62.77)
No	148 (61.67)	28 (60.87)	35 (37.23)

**Fig. 1:**
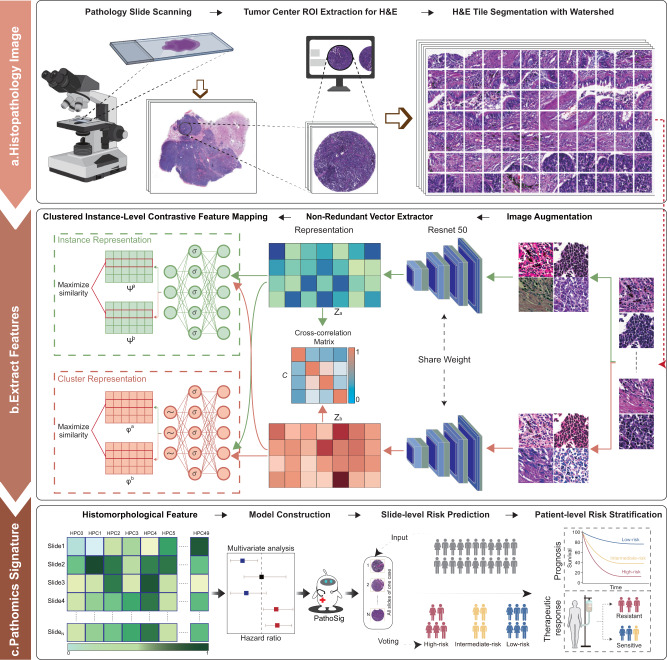
Schematic illustration of deep learning-based pathomics signature construction. **a** Segmentation of tumor regions in the whole slide image (WSI): Pathologists extracted the central regions of WSI tumors at 20x magnification, referred to as tumor tissue microarrays (TMAs). Each TMA was partitioned into non-overlapping 224 × 224 pixel tiles using a watershed algorithm. **b** Deep-learning workflow for pathological feature extraction. A deep learning framework based on unsupervised contrastive clustering was employed to extract histomorphological features from tissue pathology images. The framework consists of two main modules: a non-redundant vector extraction module and an instance-level contrastive feature mapping module. These modules automatically extract features from tissue pathology images and map them to a 2048-dimensional space, capturing unique information. The framework also enables mapping the feature space to a 50-dimensional latent space, facilitating effective image clustering. **c** Development and validation of the pathomics signature. The pathomics signature was performed using TMAs or patients, each with one or multiple TMAs. Morphological features were constructed for each TMA based on the segmented blocks and processed through clustering. The tiles within each TMA were clustered to form multiple clusters, and the proportion of each cluster relative to all clusters constituted the feature vector for that TMA. These feature vectors were utilized in Cox regression models to establish associations between tissue phenotypes and clinical annotations, enabling risk stratification of patients.

We conducted a discovery and validation multicenter study. The detailed flowchart of the study design is shown in Fig. 1 and Supplementary Figure 1. Within the CHCAMS cohort of 286 cases, there were 240 P-SCLC cases and 46 C-SCLC cases. These cases were categorized into three cohorts for the development and internal validation of the deep-learning model: the discovery cohort (*n* = 196), validation cohort-1 (P-SCLC, *n* = 44) and validation cohort-2 (C-SCLC, *n* = 46). All 94 patients in the PUCH cohort were used for external independent validation (validation cohort-3).

### Deep learning identifies histomorphological features associated with prognosis

**Fig. 2:**
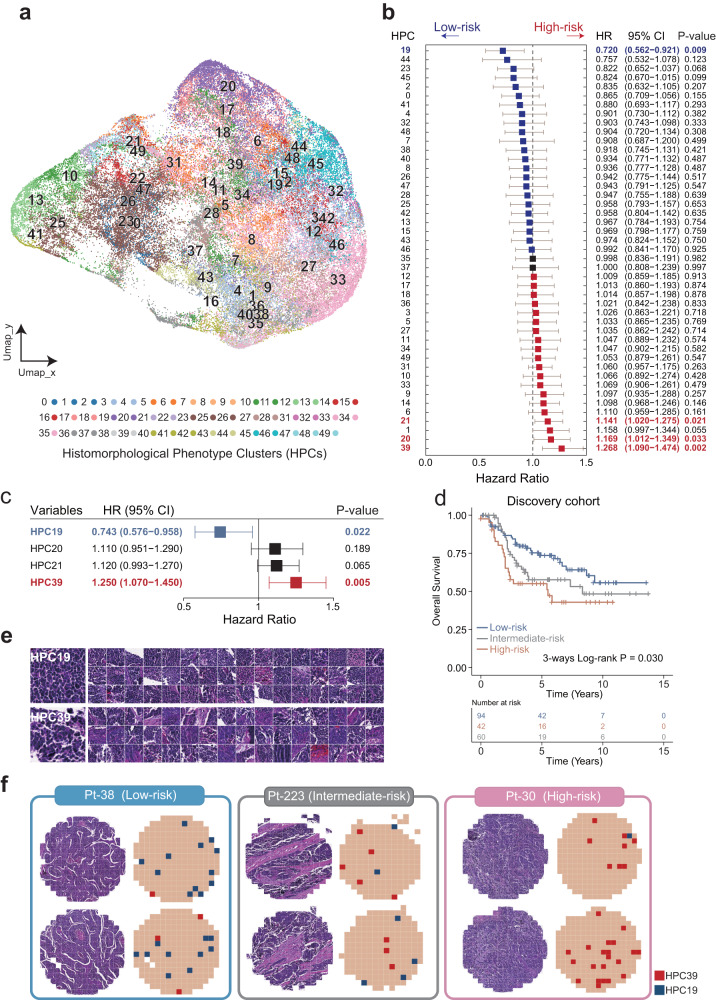
Discovery and visualization of histomorphological features associated with prognosis. **a** UMAP-based dimensionality reduction of instance-level feature vectors for each image tile, then mapping 50 tiles-level histopathological phenotype clusters (HPCs) onto the UMAP plot. **b** Forest plot visualizing the hazard ratios (95% confidence interval) of univariate Cox regression analyses of each histomorphological feature. **c** Forest plot visualizing the hazard ratios (95% confidence interval) of multivariate Cox regression analysis of four prognostic histomorphological features (HPC19, HPC20, HPC21, and HPC39). **d** Kaplan–Meier survival curves of high-risk, intermediate-risk, or low-risk groups according to the pathomics signature. **e** Visualization of histopathological phenotype clusters of HPC19 and HPC39. **f** Visualization of HPC39 and HPC19 quantities in representative TMAs for low, intermediate, and high-risk patients.

The discovery cohort was randomly divided into a training dataset (*n* = 157) and a testing dataset (*n* = 39) at a ratio of 4:1. Each H&E-stained slide was segmented into non-overlapping 224 × 224 tiles, in which tiles covering less than 60% tissue coverage were filtered out. A total of 73,199 tiles were collected for downstream analysis. Contrastive clustering was employed at both the instance- and cluster- levels to cluster the tiles from the training dataset, and 50 tile-level histomorphological phenotype clusters (HPCs) were obtained as histomorphological features, which were visualized by projecting high-dimensional data into two- dimensions using the Uniform Manifold Approximation and Projection (UMAP) (Fig. 2a). To analyze the histomorphological differences between slide block clusters, we histomorphologically selected the four farthest positions in UMAP, including upper, lower-left, lower-right and middle. We located the nearest three clusters for each position and visually inspected them (Supplementary Figure 2). We observed that greater distance between clusters corresponded to more significant morphological differences, and vice versa. This observation underscores the differential representation of slide information and morphological features in each cluster in the deep clustering of pathological slides.

To quantify the histomorphological features in each slide, we calculated the proportion of tiles belonging to each HPC relative to the total number of tiles in the slide. Univariate Cox regression analysis was performed to assess the association between histomorphological features and OS in the training dataset. Among the 50 investigated histomorphological features, we identified four histomorphological features significantly associated with OS. Of these, HPC19 was associated with improved OS (HR = 0.720, 95% CI 0.562–0.921, *p* = 0.009), while HPC20 (HR = 1.169, 95% CI 1.012–1.349, *p* = 0.033), HPC21 (HR = 1.141, 95% CI 1.020–1.275, *p* = 0.021) and HPC39 (HR = 1.268, 95% CI 1.090–1.474, *p* = 0.002) exhibited associations with poor OS (Fig. 2b). We subjected them to multivariate regression analysis to evaluate whether these four prognostic histomorphological features held independent predictive power for survival. When considering the mutual effect among four prognostic histomorphological features, this analysis revealed that only HPC19 and HPC39 showed independent predictive power for OS (Fig. 2c). Subsequently, we developed PathoSig, a composite index incorporating HPC19 and HPC39, along with the corresponding coefficients obtained from multivariate regression analysis, to predict the risk of H&E-stained slides, as follows: PathoSig = (0.2398* HPC39) + (−0.3393* HPC19).

In the testing dataset, we applied PathoSig and determined the optimal risk score threshold for H&E-stained TMA slide-level risk stratification using the five-year ROC analysis. Using this threshold and a voting algorithm, we stratified the patients in the discovery cohort into high-, intermediate-, and low-risk groups with significantly different OS (log-rank *p* = 0.030) (Fig. 2d). Notably, the predicted high-risk group demonstrated poorer OS than the low-risk group (HR = 2.055, 95% CI, 1.165–3.624; log-rank *p* = 0.011) (Fig. 2d). This observation was further supported by representative H&E-stained slides, where H&E-stained slides of high-risk patients displayed more tiles corresponding to HPC39 and fewer tiles corresponding to HPC19, relative to H&E-stained slides of low-risk patients (Fig. 2e, f).

### Prognostic significance of the PathoSig in independent validation cohorts

**Fig. 3:**
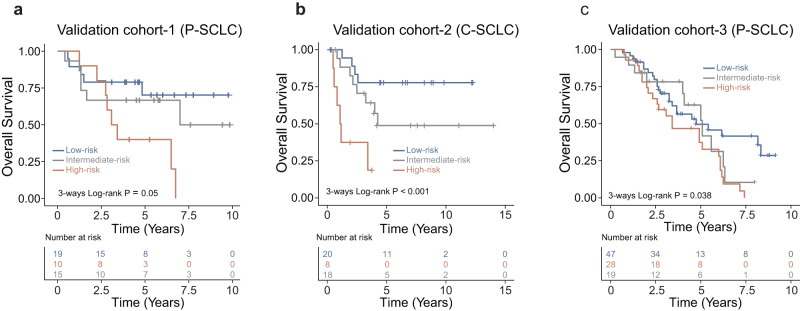
Performance validation of the pathomics signature in independent cohorts. Kaplan–Meier analysis of overall survival across low-, intermediate-, and high-risk groups in the validation-1 cohort (**a**), validation-2 cohort (**b**), and validation-3 cohort (**c**).

To validate the prognostic significance of PathoSig, we first tested it on two internal independent cohorts, validation-1(P-SCLC) and validation-2(C-SCLC), which were not used in the discovery and model training phases. Using the same PathoSig model and cutoff from the discovery cohort, we classified patients into three risk groups (low, intermediate and high) based on histomorphological phenotypes. We observed a significant stratification in OS time among the three risk groups (log-rank *p* = 0.05 and *p* < 0.001, respectively) in both internal independent cohorts (Fig. 3a-b). Kaplan–Meier survival analysis further revealed that high-risk patients had poorer OS than low-risk patients in both cohorts (validation-1 cohort: HR = 3.62, 95% CI, 1.164–11.26, *p* = 0.026; validation-2 cohort: HR = 9.478, 95% CI, 2.531–35.492, *p* = 0.001). Additionally, intermediate-risk patients displayed worse OS than low-risk patients, but better OS than high-risk patients in both validation cohorts (Fig. 3a, b). The prognostic value of PathoSig was further evaluated in the external PUCH cohort. As shown in Fig. 3c, the PathoSig successfully distinguished patients into low-, intermediate- and high-risk groups with significantly different OS (log-rank *p* = 0.038).

To examine whether PathoSig provides independent prognostic value, we conducted multivariate Cox regression analyses on PathoSig in three independent validation cohorts, incorporating various clinical features (such as sex, age, smoking history, and stage). Results from the multivariate analysis revealed that the high-risk group identified by PathoSig remained significantly associated with poor OS (validation-1 cohort: HR = 5.030, 95% CI, 1.326–19.08, *p* = 0.018; validation-2 cohort: HR = 9.960, 95% CI, 2.493–39.80, *p* = 0.001; validation-3 cohort: HR = 2.484, 95% CI, 1.336–4.615, *p* = 0.004) even after adjusting for various clinicopathological features (Table 2). These findings demonstrate that PathoSig is a robust and independent prognostic factor for predicting OS in patients with SCLC.

**Table 2:** Univariable and multivariable analyses for PathoSig and other clinical factors for overall survival in different cohorts.

Characteristics	Univariable analysis		Multivariable analysis		
	Hazard ratio (95% CI)	*P* value	Hazard ratio (95% CI)		*P* value
*Validation cohort-1 (P-SCLC)*					
PathoSig					
Low	Ref.		Ref.		
Intermediate	1.578 (0.481–5.179)	0.452	2.491 (0.701–8.859)	0.159	
High	3.620 (1.164–11.26)	**0.026**	5.030 (1.326–19.080)	**0.018**	
Sex (male vs. female)	1.135 (0.408–3.162)	0.808	1.133 (0.315–4.072)	0.848	
Age (>60 vs. ≤60)	1.816 (0.729–4.522)	0.200	7.150 (1.853–27.590)	**0.004**	
Smoking (yes vs. no)	0.698 (0.280–1.738)	0.440	0.483 (0.155–1.510)	0.211	
AJCC/UICC (III&IV vs. I&II)	2.602 (1.020–6.641)	**0.045**	3.722 (1.161–11.93)	**0.027**	
*Validation cohort-2 (C-SCLC)*					
PathoSig					
Low	Ref.		Ref.		
Intermediate	2.520 (0.756–8.403)	0.133	2.567 (0.738–8.925)	0.138	
High	9.478 (2.531–35.492)	**0.001**	9.960 (2.493–39.80)	**0.001**	
Sex (male vs. female)	0.906 (0.298–2.755)	0.862	1.419 (0.354–5.691)	0.621	
Age (>60 vs. ≤60)	1.879 (0.727–4.861)	0.193	1.966 (0.665–5.816)	0.222	
Smoking (yes vs. no)	1.865 (0.428–8.118)	0.406	1.113 (0.182–6.817)	0.908	
AJCC/UICC (III&IV vs. I&II)	1.746 (0.676–4.506)	0.249	1.931 (0.731–5.096)	0.184	
*Validation cohort-3 (P-SCLC)*					
PathoSig					
Low	Ref.		Ref.		
Intermediate	1.376 (0.665–2.847)	0.390	1.125 (0.528–2.398)	0.760	
High	2.122 (1.184–3.804)	**0.012**	2.484 (1.336–4.615)	**0.004**	
Sex (male vs. female)	0.544 (0.309–0.957)	**0.035**	0.152 (0.048–0.480)	**0.001**	
Age (>60 vs. ≤60)	1.277 (0.765–2.132)	0.349	1.242 (0.723–2.135)	0.432	
Smoking (yes vs. no)	0.681 (0.372–1.248)	0.214	3.828 (1.180–12.420)	**0.025**	
AJCC/UICC (III&IV vs. I&II)	2.194 (1.256–3.834)	**0.006**	1.919 (1.066–3.455)	**0.030**	

Bold values indicates a significant *p* value.

### Predictive value of pathomics signature for therapeutic response

**Fig. 4:**
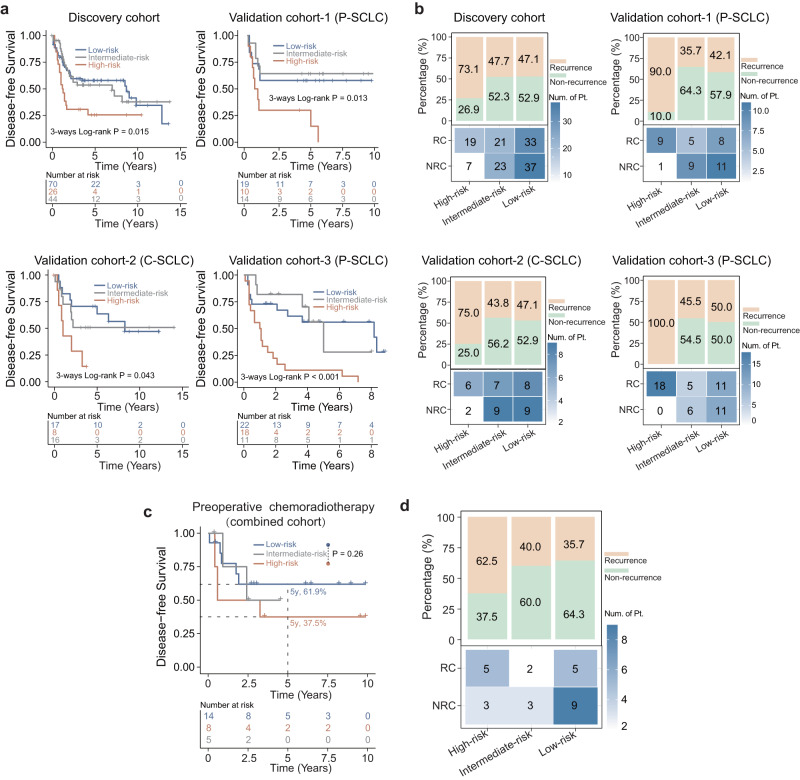
Association between pathomics signature and the therapeutic benefits from postoperative or preoperative chemoradiotherapy. **a** The Kaplan–Meier analysis of disease-free survival in patients who underwent postoperative chemoradiotherapy for the low-, intermediate-, and high-risk groups in different cohorts. **b** The percentage histogram showing the recurrence and non-recurrence proportion in low-, intermediate-, and high-risk groups for patients who underwent postoperative chemoradiotherapy in different cohorts. **c** The Kaplan–Meier analysis of disease-free survival in patients who received preoperative chemoradiotherapy for low-, intermediate-, and high-risk groups in a combined cohort. **d** The percentage histogram showing the recurrence and non-recurrence proportion in low-, intermediate-, and high-risk groups for patients who received preoperative chemoradiotherapy in a combined cohort.

The predictive value of the pathomics signature for the clinical efficacy of chemoradiotherapy was evaluated by analyzing DFS and disease recurrence rates in different cohorts. In all four cohorts, patients who received chemoradiotherapy after surgery showed significantly shorter DFS durations when classified as high-risk by PathoSig, compared to the low- and intermediate-risk groups (log-rank *p* = 0.015 for discovery cohort; *p* = 0.013 for validation-1 cohort; *p* = 0.043 for validation-2 cohort and *p* < 0.001 for validation-3 cohort) (Fig. 4a). In addition, the high-risk group consistently displayed higher recurrence rates (73.1%, 75%, 90%, and 100%) compared to the low-risk (47.1%, 43.8%, 42.1%, and 50%) and intermediate-risk groups (47.7%, 47.1%, 35.7%, and 45.5%) across all four cohorts (Fig. 4b). Multivariate Cox analysis also indicated the independent prognostic value of PathoSig for DFS when adjusting for various clinical features (Discovery cohort: HR = 1.989, 95% CI, 1.119–3.538, *p* = 0.019; validation-1 cohort: HR = 3.755, 95% CI, 1.213–11.62, *p* = 0.022; validation-2 cohort: HR = 3.464, 95% CI, 1.055–11.38, *p* = 0.041 and validation-3 cohort: HR = 2.626, 95% CI, 1.462–4.714, *p* = 0.001) (Table 3). For patients with SCLC who underwent preoperative chemoradiotherapy, four cohorts were combined for further analysis due to the limitation of a small number of patients in each cohort. The high-risk group was associated with shorter DFS durations (Fig. 4c). The 5-year DFS rate for the high-risk group was 37.5%, whereas the corresponding rate for the low-risk group was 61.9%, although statistical significance was not reached, likely due to sample size limitations (log-rank *p* = 0.26) (Fig. 4c). Additionally, the high-risk group had an increased risk of recurrence compared to the low- and intermediate-risk groups (62.5% vs. 40% and 35.7%) (Fig. 4d).

**Table 3:** Univariable and multivariable analyses for PathoSig and other clinical factors for disease-free survival in different cohorts.

Characteristics	Univariable analysis		Multivariable analysis	
	Hazard ratio (95% CI)	*P* value	Hazard ratio (95% CI)	*P* value
*Discovery cohort (P-SCLC)*				
PathoSig				
Low	Ref.		Ref.	
Intermediate	1.031 (0.596–1.782)	0.914	1.130 (0.637–2.004)	0.676
High	2.172 (1.228–3.842)	**0.008**	1.989 (1.119–3.538)	**0.019**
Sex (male vs. female)	1.861 (1.080–3.207)	**0.025**	1.366 (0.673–2.773)	0.388
Age (>60 vs. ≤60)	0.670 (0.406–1.107)	0.118	0.830 (0.493–1.398)	0.483
Smoking (Yes vs. No)	1.635 (0.976–2.737)	0.062	1.281 (0.656–2.502)	0.469
AJCC/UICC (III&IV vs. I&II)	2.314 (1.442–3.712)	**0.001**	2.127 (1.301–3.478)	**0.003**
*Validation cohort–1 (P–SCLC)*				
PathoSig				
Low	Ref.		Ref.	
Intermediate	0.771 (0.252–2.358)	0.648	1.045 (0.317–3.452)	0.942
High	2.965 (1.133–7.761)	**0.027**	3.755 (1.213–11.62)	**0.022**
Sex (Male vs. Female)	1.292 (0.506–3.304)	0.592	1.521 (0.458–5.048)	0.494
Age ( > 60 vs. ≤60)	1.189 (0.484–2.917)	0.706	1.992 (0.667–5.951)	0.217
Smoking (Yes vs. No)	1.001 (0.432–2.321)	0.997	0.633 (0.221–1.812)	0.394
AJCC/UICC (III&IV vs. I&II)	2.403 (1.006–5.736)	**0.048**	1.855 (0.725–4.750)	0.198
*Validation cohort-2 (C-SCLC)*				
PathoSig				
Low	Ref.		Ref.	
Intermediate	1.328 (0.476–3.708)	0.588	1.179 (0.394–3.527)	0.768
High	3.751 (1.233–11.415)	**0.020**	3.464 (1.055–11.38)	**0.041**
Sex (male vs. female)	0.749 (0.274–2.046)	0.573	0.933 (0.254–3.427)	0.917
Age (>60 vs. ≤60)	1.466 (0.619–3.468)	0.384	1.414 (0.496–4.032)	0.517
Smoking (yes vs. no)	1.489 (0.437–5.075)	0.525	1.153 (0.264–5.036)	0.850
AJCC/UICC (III&IV vs. I&II)	1.215 (0.515–2.870)	0.657	1.144 (0.476–2.749)	0.763
*Validation cohort-3 (P-SCLC)*				
PathoSig				
Low	Ref.		Ref.	
Intermediate	1.255 (0.631–2.499)	0.517	1.305 (0.652–2.613)	0.452
High	2.314 (1.330–4.023)	**0.003**	2.626 (1.462–4.714)	**0.001**
Sex (male vs. female)	0.755 (0.440–1.297)	0.309	0.447 (0.153–1.307)	0.141
Age (>60 vs. ≤60)	1.005 (0.617–1.638)	0.983	1.010 (0.618–1.650)	0.968
Smoking (Yes vs. No)	0.802 (0.449–1.433)	0.456	1.620 (0.524–5.014)	0.402
AJCC/UICC (III&IV vs. I&II)	1.887 (1.099–3.237)	**0.021**	1.823 (1.041–3.193)	**0.036**

Bold values indicates a significant *p* value.

### PathoSig added value to the current staging system

**Fig. 5:**
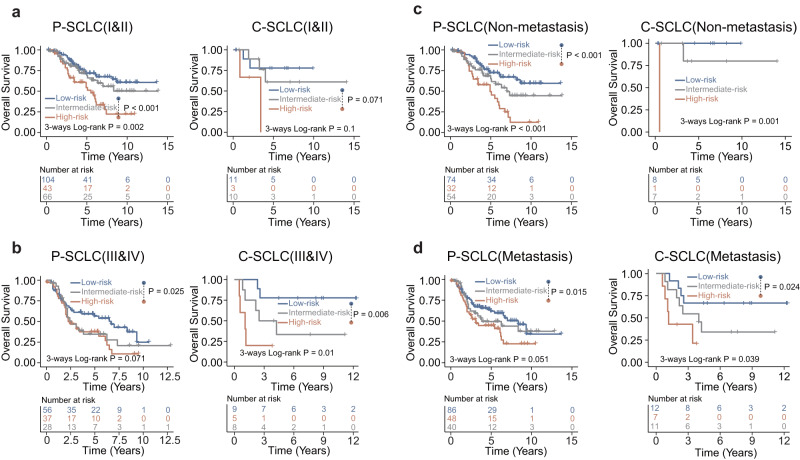
The performance evaluation of pathomics signature in the current staging system. **a** The Kaplan–Meier analysis of overall survival in the low-, intermediate-, and high-risk groups for stage I&II patients with P-SCLC or C-SCLC. **b** The Kaplan–Meier analysis of overall survival in the low-, intermediate-, and high-risk groups for stage III&IV patients with P-SCLC or C-SCLC. **c** The Kaplan–Meier analysis of overall survival in the low-, intermediate-, and high-risk groups for patients with non-metastatic lymph nodes. **d** The Kaplan–Meier analysis of overall survival in the low-, intermediate-, and high-risk groups for patients with metastatic lymph nodes.

To assess whether PathoSig can provide improved survival predictions within the same clinical stage, we carried out a stratified analysis of SCLC patients with early-stage (stage I/II) and late-stage (stage III/IV) disease for both P-SCLC and C-SCLC patients, respectively. Our findings indicate that PathoSig can potentially refine existing stage-based prognoses in SCLC. Kaplan–Meier survival analysis revealed that PathoSig could classify early-stage patients into high-, low- and intermediate-risk groups, with obvious differences in OS and DFS observed between the high- and low-risk groups in both P-SCLC (log-rank *p* < 0.001 for both) and C-SCLC patients (log-rank *p* = 0.071 and 0.018, respectively) (Fig. 5a and Supplementary Figure 3a). Similarly, PathoSig demonstrated significant prognostic value for OS and DFS in late-stage patients, both in P-SCLC (log-rank *p* = 0.025 and 0.007, respectively) and C-SCLC patients (log-rank *p* = 0.006 and 0.16, respectively) (Fig. 5b and Supplementary Figure 3b). We further conducted a stratified analysis of patients with or without metastasis and found that PathoSig exhibited prognostic significance in both metastatic and non-metastatic subgroups of patients (Fig. 5c, d and Supplementary Figure 3c, d). In the non-metastatic subgroup of patients, high-risk PathoSig was associated with significantly shorter OS and DFS compared to intermediate- and low-risk PathoSig in the P-SCLC cohort (log-rank *p* < 0.001 for OS and *p* < 0.001 for DFS) and the C-SCLC cohort (log-rank *p* = 0.001 for OS and log-rank *p* = 0.79 for DFS) (Fig. 5c and Supplementary Figure 3c). Similarly, in the metastatic subgroup, samples with high-risk PathoSig also had poorer OS and DFS compared to those with intermediate- and low-risk PathoSig in both P-SCLC (log-rank *p* = 0.051 for OS and *p* = 0.0065 for DFS) and C-SCLC (log-rank *p* = 0.039 for OS and *p* = 0.15 for DFS) patients (Fig. 5d and Supplementary Figure 3d). These results collectively suggest that PathoSig can add additional prognostic value to the current staging system.

### Stratification analysis of PathoSig for molecular subtypes

**Fig. 6:**
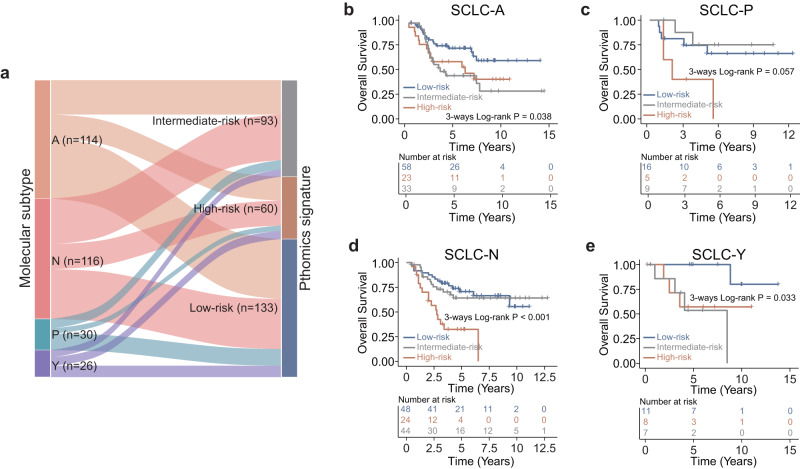
Stratification analysis of pathomics signature based on transcription factors-defined molecular subtypes (SCLC-A, SCLC-N, SCLC-P, and SCLC-Y). **a** Alluvial plot showing the distribution of molecular subtypes according to the pathomics signature. Kaplan–Meier analysis of overall survival in the low-, intermediate-, and high-risk groups for patients with SCLC-A (**b**), SCLC-P (**c**), SCLC-N (**d**), and SCLC-Y (**e**).

We further investigated the association between PathoSig and consensus molecular subtypes defined by the predominant expression of transcription factors ASCL1 (SCLC-A), NEUROD1 (SCLC-N), POU2F3 (SCLC-P) and YAP1 (SCLC-Y)^11^. We measured the protein expression of ASCL1, NEUROD1, POU2F3, and YAP1 by immunohistochemistry in 286 SCLC patients of the CHCAMS cohort, and then classified these SCLC patients into one of four subtypes based on the predominant expression of four transcription factors. Notably, 50.9% of SCLC-A subtype patients were classified into the low-risk group based on our PathoSig, while the high-risk group exhibited the highest proportion of patients with SCLC-N subtype (40.0%) (Fig. 6a). Furthermore, we conducted a survival risk stratification analysis by integrating the four molecular subtypes with PathoSig. Notably, patients with the same molecular subtype were classified into different risk groups with different OS and DFS outcomes (log-rank *p* = 0.038 and 0.095 for SCLC-A subtype; *p* = 0.057 and 0.15 for SCLC-P subtype; *p* < 0.001 and 0.001 for SCLC-N subtype; *p* = 0.033 and 0.064 for SCLC-Y subtype) (Fig. 6b–e and Supplementary Figure 4). These findings indicate that PathoSig was able to further stratify patients with different molecular subtypes, providing more comprehensive prognostic information beyond the molecular subtypes themselves.

## Discussion

SCLC presents unique challenges in prognosis prediction and treatment compared to other lung cancer types^12^. Unlike lung adenocarcinoma, where molecular subtyping and targeted therapies have shown promise, SCLC is often diagnosed at advanced stages, making surgical intervention less feasible. Additionally, the limited availability of clinical pathological tissue samples presents a significant obstacle to in-depth research on SCLC^13,14^, and restricts the application of traditional methods that rely on biopsied tissues and molecular experiments to understand tumor characteristics and vulnerabilities in SCLC^15,16^.

This study addressed these challenges by leveraging deep learning techniques and H&E-stained histopathology images to develop PathoSig, a predictive pathomics signature for prognosis and therapeutic response in SCLC. We introduced an unbiased method for histomorphological phenotype representation through self-supervised learning and community detection. Self-supervised learning offers independence from manual labeling or delineation of target regions, reducing the potential bias introduced by human sampling and saving time. Furthermore, we concentrated on pixel tile segmentation and proposed an unbiased approach for extracting histomorphological phenotype representations^17,18^. This method divides the slides into multiple non-overlapping mosaic-like regions, providing supplementary information on cellular arrangement and histological texture characteristics. Importantly, it eliminates the need to retrain the model, as would be necessary with supervised or weakly-supervised end-to-end solutions.

The validation of PathoSig in both medical center cohorts demonstrated its robust prognostic value and significant potential for clinical applications. The stratification of patients into low-, intermediate-, and high-risk groups based on PathoSig allowed for significant differentiation in OS and DFS, providing more precise predictions of patient outcomes. Furthermore, the prognostic capability of PathoSig extends not only to P-SCLC but also to C-SCLC, a highly heterogeneous subgroup that has been relatively understudied in previous research. Additionally, PathoSig accurately predicted the clinical efficacy of post-surgery chemoradiotherapy in patients. We specifically focused on two significant indicators, DFS and disease recurrence rates, to evaluate the predictive efficacy of PathoSig. To avoid the confounding effects of neoadjuvant therapy, we divided patients into “surgery-sequential chemoradiotherapy” and “chemoradiotherapy-sequential surgery” to verify the treatment effects of different risk groups. The results confirm that PathoSig remains valuable in predicting treatment responses, even when patients have received neoadjuvant therapy, highlighting its potential as a tool for identifying high-risk patients from SCLC postoperative pathological sections, an important aspect of postoperative management and supplementary treatment planning to enhance DFS and reduce recurrence rates.

Additionally, PathoSig has demonstrated significant prognostic stratification capabilities for the recently proposed transcription factor-based molecular subtypes^11^. While preclinical studies indicate that the subtypes may have distinct treatment vulnerabilities^4^, their prognostic significance in clinical tumor sample-based studies remains controversial^19,20^. Qi et al. reported that the YAP1 and ASCL1 subtypes showed the best and worst prognosis, respectively^19^, but most other studies have failed to confirm the prognostic stratification significance of these molecular subtypes^21,22^. Our earlier research found that the SCLC-Y subtype has a poorer prognosis in C-SCLC, while its prognostic significance remains unclear in P-SCLC^21^, highlighting the need to investigate further and validate these molecular subtypes to determine their prognostic significance in clinical settings. Nevertheless, our findings indicate that PathoSig can provide comprehensive prognostic information beyond molecular subtyping, suggesting its potential to improve risk stratification and guide treatment decisions for patients with SCLC.

Despite the promising results obtained in our study, it is important to acknowledge several limitations. Firstly, the retrospective nature of our study and the reliance on surgical resection samples raise concerns about the generalizability of PathoSig for extensive stage cases, which mostly rely on biopsies. Further validation using biopsy tissue samples is necessary to establish the validity of our findings. Secondly, the slide-level risk stratification rules used in this study seem too rigid, lacking a nuanced approach rather than a clear-cut label due to the intratumoral and intertumoral heterogeneity of SCLC. More sophisticated models or risk stratification strategies should be introduced to handle these heterogeneities more accurately. Moreover, obtaining the necessary medical licensing and regulatory approvals may present challenges for translating the deep learning model into routine clinical practice.

In conclusion, our study highlights the potential of utilizing histopathology images-based deep learning to improve prognostic predictions and therapeutic response evaluation in SCLC. The PathoSig we developed, validated through extensive analysis of multicenter retrospective datasets, demonstrates remarkable predictive performance, robustness and generalizability, offering clinicians valuable insights for making informed treatment decisions. Further validation studies and integration of PathoSig into clinical practice are warranted to fully realize its potential in improving patient outcomes in SCLC.

## Methods

### Ethics statement

This multicenter retrospective study has received ethical approval from the Ethics Committee and Institutional Review Boards of the Cancer Hospital, Chinese Academy of Medical Science (No. 22/250-3452) and Peking University Cancer Hospital (No. 2023KT23). As this was a retrospective study, the requirement for informed consent was waived.

### Study participants and patient cohorts

We retrospectively collected 380 surgically resected and pathologically confirmed specimens of SCLC from two independent medical centers, including 286 patients from the Cancer Hospital, Chinese Academy of Medical Science (CHCAMS cohort), spanning the period from January 2005 to December 2016, and 94 patients from the Peking University Cancer Hospital between January 2010 and April 2023 (PUCH cohort). The inclusion criteria for the study were as follows: (i) Pathologically diagnosed with SCLC, including pure SCLC or combined SCLC; (ii) Availability of complete clinical and pathologic information; (iii) Availability of follow-up data for both disease-free survival (DFS) and overall survival (OS), and (iv) Accessible tumor tissues. DFS is defined as the time from primary surgery to the first confirmed tumor recurrence, progression, death, or the last follow-up for disease-free patients. OS is defined as the time from the surgery date to death or the last follow-up. The clinical characteristics of these two cohorts are shown in Table 1.

### Acquisition of H&E-stained histopathology images

Archival formalin-fixed paraffin-embedded (FFPE) tumor sections were retrieved from the pathological specimen repository of CHCAMS and PUCH cohorts, and subsequently reviewed by experienced thoracic pathologists following the diagnostic criteria of the 2021 World Health Organization classification of lung tumors^23^. For cases with atypical morphological features, neuroendocrine markers such as Neural Cell Adhesion Molecule 1 (NCAM1, also known as CD56), Synaptophysin (Syn) and Chromogranin A (ChrA), and proliferative index of Ki-67 were used to differentiate poorly differentiated squamous cell carcinomas, adenocarcinomas, carcinoid and atypical carcinoid.

Next, representative tissue slides and corresponding tumor blocks were chosen for constructing tissue microarrays (TMAs) (1–4 cores per case, 1.5 mm in diameter and 6 mm in depth). Consecutive tumor sections with a thickness of 4 μm were obtained for H&E staining using a fully automated and intelligent staining and sealing system (Dakewe Biotech Co., Ltd. Shenzhen, China). Again, experienced thoracic pathologists confirmed qualified tissue core slides with more than 60% tumor purity for downstream digital scanning captured at 20x magnification using a digital slide scanner (KF-LPE-006, Jiangfeng Biotechnology Co., Ltd., China). We obtained 573 TMA slides from 286 patients of the CHCAMS cohort and 188 TMA slides from 94 patients of the PUCH cohort for subsequent image preprocessing.

### Image preprocessing

We initially segmented the TMA slides captured at 20x magnification into non-overlapping 224 × 224 pixel tiles. Otsu thresholding was applied to effectively separate the white background from the tissue regions within the tiles, ensuring that only tiles with tissue coverage surpassing 60% of the total area were retained. To enhance the robustness of our model, we employed six random image augmentation techniques, namely flipping, rotation, contrast adjustment, scaling, HSV adjustment and noise addition. These augmentation techniques were applied during the preprocessing stage, resulting in an increased diversity of the training dataset. We generated 223,002 tiles for two medical center cohorts through this image preprocessing process.

### Extraction of histomorphological features via self-supervised deep learning architecture

We proposed a self-supervised deep learning framework called DL-CC (Deep Learning with Contrastive Clustering) to extract histomorphological features from histopathological images. The DL-CC framework consists of two main modules: the Non-Redundant Vector Extractor module and the Clustered Instance-Level Contrastive Feature Mapping module. These modules automatically extract features from histopathological images and map them into a 2048-dimensional space, capturing unique information. Additionally, the framework allows for mapping the feature space to a 50-dimensional entity space, enabling effective image clustering.

For the Non-Redundant Vector Extractor module, we utilized a pair of ResNet50 networks with shared weights to handle distinct augmented images, allowing us to capture complex morphological features within the tissue tiles. The resulting features are then mapped into a 2048-dimensional space, effectively converting each 224 × 224 pixel image into a tile vector representation $\{{\rm{z}}\in {{\mathbb{R}}}^{d},{\rm{d}}=2048\}$. To capture robust feature vectors, a comprehensive strategy is employed to minimize the total loss. Specifically, the diagonal loss is employed to define the scaling and rotation boundaries of the feature vectors. Simultaneously, the off-diagonal loss is utilized to control the orthogonality of vectors. The cross-correlation matrix c is calculated in Eq. (1):

1 $${\rm{c}}=\frac{1}{d}{{BN}\left({z}_{a}\right)}^{T}\bullet {BN}\left({z}_{b}\right)$$

Here, ${z}_{a}$ and ${z}_{b}$ represent two feature vectors extracted by ResNet50 after image augmentation, d represents the dimensionality of the tile vector representation, and BN represents the Batch Normalization operation.

The representation loss is then calculated in Eq. (2):

2 $${\rm{Representation\; loss}}=\mathop{\sum }\limits_{i=1}^{d}{{(c}_{{ii}}-1)}^{2}+{\rm{\lambda }}\mathop{\sum }\limits_{i-1}^{d-1}\mathop{\sum }\limits_{j=i+1}^{d}{c}_{{ij}}^{2}$$

Where $c$ represents the correlation matrix, and ${\rm{\lambda }}$ represents the weight of the loss for uncorrelatedness.

For Clustered Instance-Level Contrastive Feature Mapping module, two strategies are employed in the clustering part of our model: instance-level and cluster-level contrastive heads. The instance-level contrastive head is designed to optimize the representation of images in the feature space by maximizing the similarity of ‘positive pairs’ (generated from the same tile images but subjected to different augmentation techniques) and minimizing the similarity of ‘negative pairs’ (generated from different tile images). The cluster-level contrastive head projects the morphological features of the image into a 50-dimensional feature vector, serving as a “soft label” for each tile and indicating the probabilistic degree of belongingness to a specific class.

The instance-level contrastive loss is calculated in Eq. (3):

3 $${Instance}\,{loss}=\frac{1}{\left(2N\right)}\mathop{\sum }\limits_{i=1}^{N}\left({\varphi }_{i}^{a}+{\varphi }_{i}^{b}\right)$$

The cluster-level contrastive loss is calculated in Eq. (4):

4 $${Cluster}\,{loss}=\frac{1}{\left(2M\right)}\mathop{\sum }\limits_{i=1}^{M}\left({{\rm{\psi }}}_{i}^{a}+{{\rm{\psi }}}_{i}^{b}\right)-H\left(Y\right)$$

Here, N represents the batch size (number of image tiles processed in each iteration), M denotes the number of clusters (distinct groups the image tiles are mapped to), and H(Y) is the entropy of cluster assignment probabilities. These loss functions enhance the model’s ability to learn discriminative feature representations and improve clustering performance.

Our approach simultaneously optimizes three loss components, namely the Representation loss, instance-level contrastive loss and cluster-level contrastive loss, to achieve the overall objective function^24^, which can be expressed in Eq. (5):

5 $$\begin{array}{l}{Loss}=\left({Instance}\,{loss}+{Cluster}\,{loss}\right)* \left(1-\alpha \right)\\\qquad\quad+\,{\rm{Representation}}\; {\rm{loss}}* {{\alpha }}\end{array}$$

Here, the hyperparameter α is specifically introduced to regulate the balance between the Representation loss and the instance-level and cluster-level contrastive losses in our proposed methodology. It allows for controlling the relative importance of each loss component.

The DL-CC framework enables a precise transformation of image data from each slide into an abstract representation in the histomorphological feature space. The resulting vector representations for each H&E-stained histopathological image are high-dimensional and correspond to the number of histomorphological clusters identified. Each vector dimension represents the relative proportion of that cluster within the histopathological image, providing information about the composition and distribution of different histomorphological features within the image.

### Development of a pathomics signature (PathoSig)

The univariate analysis was used to assess the relationship between individual histomorphological features and survival time, and histomorphological features that are statistically significantly associated with overall survival are evaluated independently for their prognostic significance in the multivariate analysis. A PathoSig was constructed as a linear combination of the selected prognostic histomorphological features based on the estimated regression coefficients obtained from the multivariate analysis. To determine the risk label for each H&E-stained TMA slide, a threshold is set using the optimal risk score determined from the five-year ROC analysis in the testing dataset. H&E-stained TMA slides with risk scores above the threshold are classified as high-risk, while those below the threshold are classified as low-risk. For patient-level risk stratification, a voting strategy is employed to aggregate the risk assessments. If all H&E-stained TMA slides from a patient are consistently classified as either high-risk or low-risk, the patient is predicted to belong to the corresponding risk group. However, in cases where the H&E-stained TMA slides derived from one patient showed conflicting risk features (both high-risk and low-risk), the patient was classified as an intermediate-risk group.

### Statistical analysis

All statistical analyses were performed using R software (version 4.1.3) and relevant R packages. Continuous variables between two groups are compared using a Wilcoxon rank sum test, while categorical variables are compared using Fisher’s exact test or the Chi-squared test. Survival curves were generated using the Kaplan–Meier method, and the log-rank test was employed to compare the curves using the R package ‘survminer’ (version 0.4.9). Cox regression analysis was conducted for univariate and multivariate analyses to estimate the hazard ratios (HR) and corresponding 95% confidence intervals (CI).

### Reporting summary

Further information on research design is available in the Nature Research Reporting Summary linked to this article.

### Supplementary information

Supplementary files

Reporting Summary

## Data Availability

The H&E images and clinical information analyzed during the current study are not publicly available for patient privacy purposes. Data access can be obtained through a reasonable request to L.Y. (yanglin@cicams.ac.cn). Access to the data will be restricted to non-commercial research, which removes patient-sensitive information.

## Code availability

The source code of this work can be downloaded from https://github.com/ZhoulabCPH/PathoSig.
