# Supplementary material for: Histopathology images-based deep learning prediction of prognosis and therapeutic response in small cell lung cancer
Source: NPJ Digit Med. 2024 Jan 18;7:15. doi: 10.1038/s41746-024-01003-0 (PMC10796367; doi:10.1038/s41746-024-01003-0)
Supplement: Supplementary file 1 — Supplementary files [file 41746_2024_1003_MOESM1_ESM.pdf]

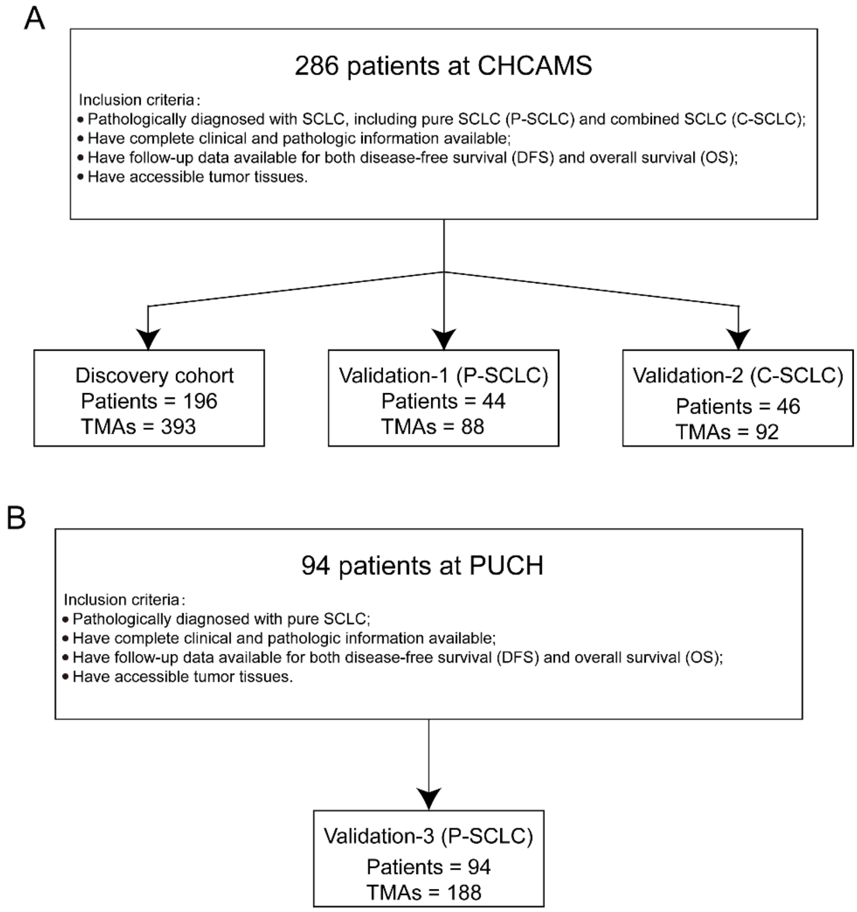


**Supplementary Figure 1.** Flowchart of the study design. All 240 P-SCLC patients in the CHCAMS cohort were randomly divided into a discovery cohort (n=196) for model development and an independent internal validation cohort (validation-1 cohort, n=44). 46 C-SCLC patients in the CHCAMS cohort were used as another internal validation cohort (validation-2 cohort, n=46). The PUCH cohort was used for external validation cohort (validation-3 cohort, n=94).

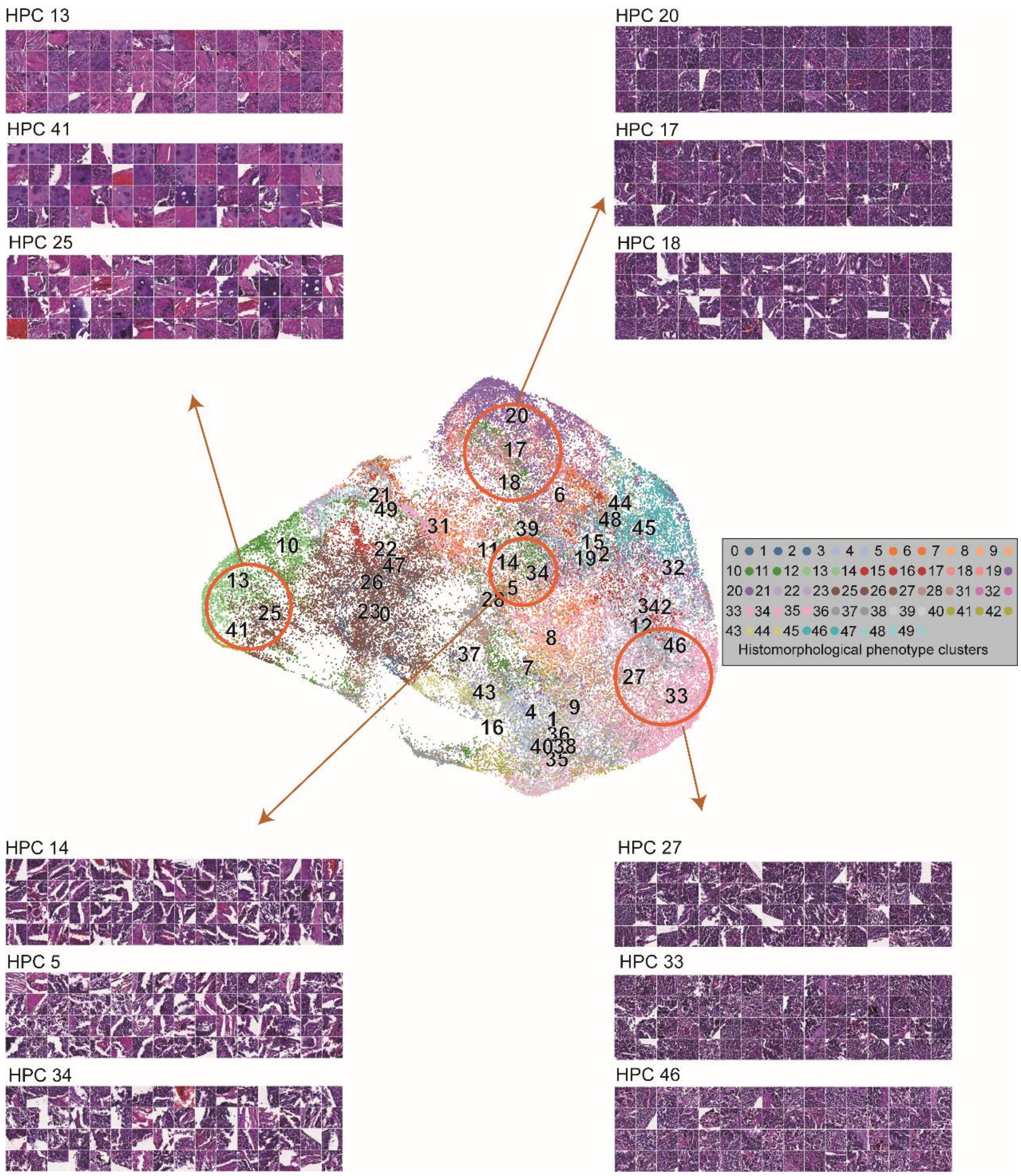


**Supplementary Figure 2.** UMAP analysis of four positions reveals distance and morphological differences among image tile clusters.

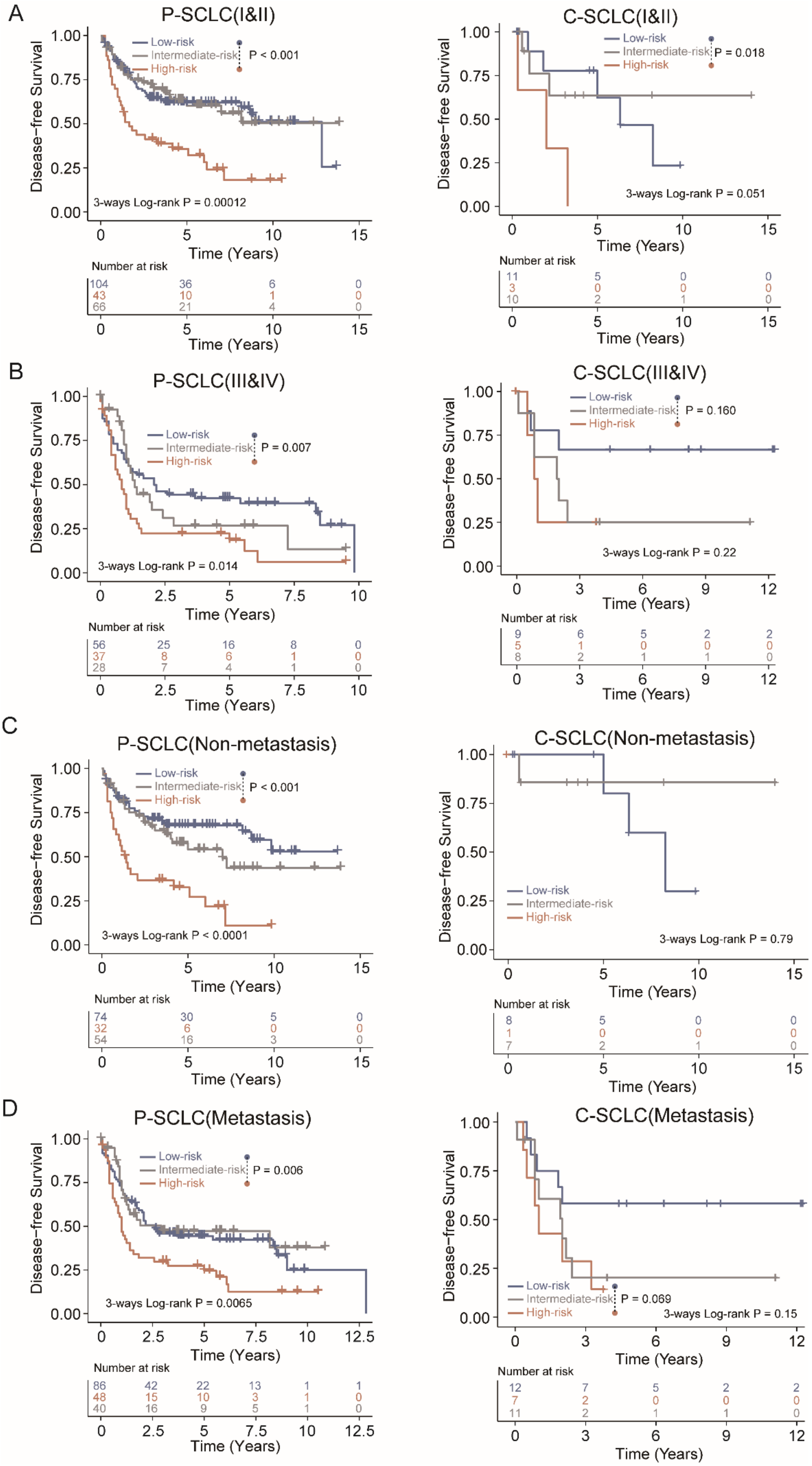
A
P-SCLC(I&II)
Disease−free Survival
Low-risk
Intermediate-risk
High-risk
P < 0.001
3-ways Log-rank P = 0.00012
Time (Years)
Number at risk
104 36 6 0
43 10 1 0
66 21 4 0
C-SCLC(I&II)
Low-risk
Intermediate-risk
High-risk
P = 0.018
3-ways Log-rank P = 0.051
Number at risk
11 5 0 0
3 0 0 0
10 2 1 0
B
P-SCLC(III&IV)
Low-risk
Intermediate-risk
High-risk
P = 0.007
3-ways Log-rank P = 0.014
Number at risk
56 25 16 8 0
37 8 6 1 0
28 7 4 1 0
C-SCLC(III&IV)
Low-risk
Intermediate-risk
High-risk
P = 0.160
3-ways Log-rank P = 0.22
Number at risk
9 6 5 2 2
5 1 0 0 0
8 2 1 1 0
C
P-SCLC(Non-metastasis)
Low-risk
Intermediate-risk
High-risk
P < 0.001
3-ways Log-rank P < 0.0001
Number at risk
74 30 5 0
32 6 0 0
54 16 3 0
C-SCLC(Non-metastasis)
Low-risk
Intermediate-risk
High-risk
3-ways Log-rank P = 0.79
Number at risk
8 5 0 0
1 0 0 0
7 2 1 0
D
P-SCLC(Metastasis)
Low-risk
Intermediate-risk
High-risk
P = 0.006
3-ways Log-rank P = 0.0065
Number at risk
86 42 22 13 1 1
48 15 10 3 1 0
40 16 9 5 1 0
C-SCLC(Metastasis)
Low-risk
Intermediate-risk
High-risk
P = 0.069
3-ways Log-rank P = 0.15
Number at risk
12 7 5 2 2
7 2 0 0 0
11 2 1 1 0

**Supplementary Figure 3. The therapeutic benefits of the pathomics signature in the current staging system. (A)** The Kaplan-Meier analysis of disease-free survival in the low-, intermediate-, and high-risk groups for stage I&II patients with P-SCLC or C-SCLC. (**B)** The Kaplan-Meier analysis of disease-free survival in the low-, intermediate-, and high-risk groups for stage III&IV patients with P-SCLC or C-SCLC. (**C)** The Kaplan-Meier analysis of disease-free survival in the low-, intermediate-, and high-risk groups for patients with non-metastatic lymph nodes. (D) The Kaplan-Meier analysis of disease-free survival in the low-, intermediate-, and high-risk groups for patients with metastatic lymph nodes.

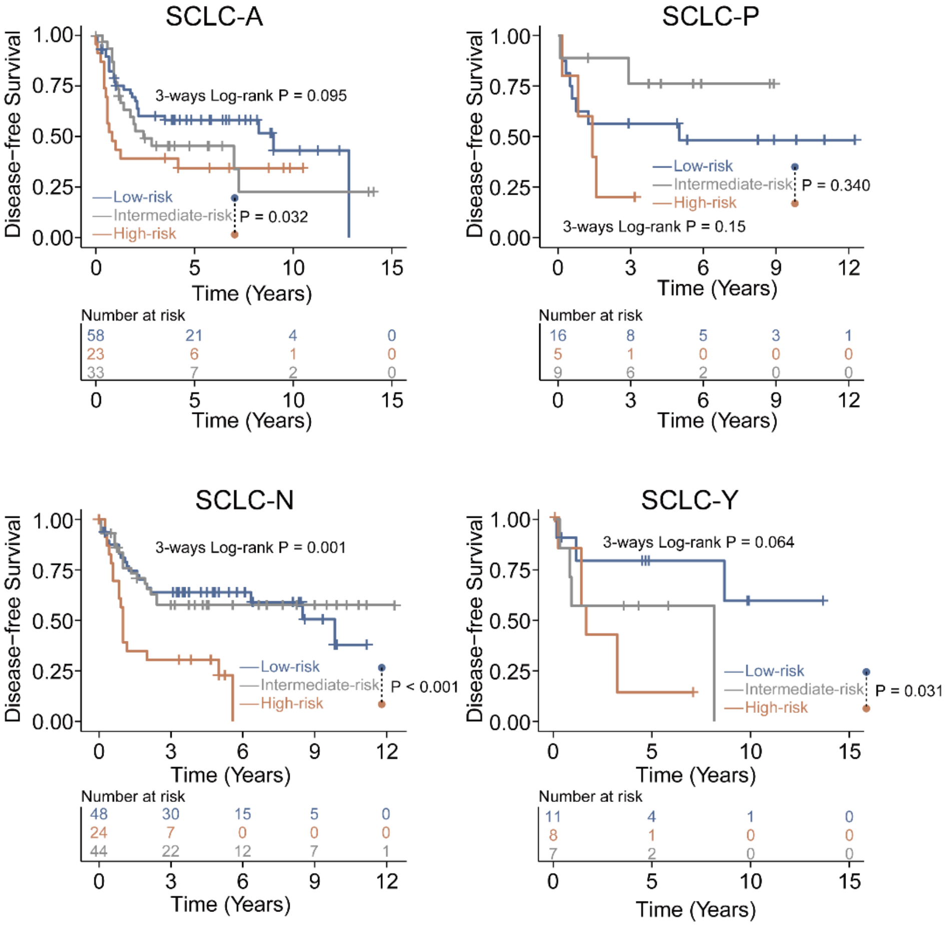


**Supplementary Figure 4.** Kaplan-Meier analysis of disease-free survival in the low-, intermediate-, and high-risk groups for patients with SCLC-A, SCLC-P, SCLC-N, and SCLC-Y subtypes.
