# Supplementary material for: Histopathology images-based deep learning prediction of prognosis and therapeutic response in small cell lung cancer
Source: NPJ Digit Med. 2024 Jan 18;7:15. doi: 10.1038/s41746-024-01003-0 (PMC10796367; doi:10.1038/s41746-024-01003-0)
Supplement: Supplementary file 2 — Reporting Summary [file 41746_2024_1003_MOESM2_ESM.pdf]

nature portfolio

Corresponding author(s): Meng Zhou, Lin Yang， Dongmei Lin

Last updated by author(s): Jul 13, 2023

Reporting Summary

Nature Portfolio wishes to improve the reproducibility of the work that we publish. This form provides structure for consistency and transparency in reporting. For further information on Nature Portfolio policies, see our Editorial Policies and the Editorial Policy Checklist.

Statistics

For all statistical analyses, confirm that the following items are present in the figure legend, table legend, main text, or Methods section.

n/a	Confirmed	
☐	☒	The exact sample size (*n*) for each experimental group/condition, given as a discrete number and unit of measurement
☐	☒	A statement on whether measurements were taken from distinct samples or whether the same sample was measured repeatedly
☐	☒	The statistical test(s) used AND whether they are one- or two-sided<br>*Only common tests should be described solely by name; describe more complex techniques in the Methods section.*
☒	☐	A description of all covariates tested
☒	☐	A description of any assumptions or corrections, such as tests of normality and adjustment for multiple comparisons
☐	☒	A full description of the statistical parameters including central tendency (e.g. means) or other basic estimates (e.g. regression coefficient) AND variation (e.g. standard deviation) or associated estimates of uncertainty (e.g. confidence intervals)
☐	☒	For null hypothesis testing, the test statistic (e.g. *F*, *t*, *r*) with confidence intervals, effect sizes, degrees of freedom and *P* value noted<br>*Give P values as exact values whenever suitable.*
☒	☐	For Bayesian analysis, information on the choice of priors and Markov chain Monte Carlo settings
☒	☐	For hierarchical and complex designs, identification of the appropriate level for tests and full reporting of outcomes
☒	☐	Estimates of effect sizes (e.g. Cohen's *d*, Pearson's *r*), indicating how they were calculated

Our web collection on statistics for biologists contains articles on many of the points above.

Software and code

Policy information about availability of computer code

Data collection	We retrospectively collected 380 surgically resected and pathologically confirmed specimens of SCLC from two independent medical centers, including 286 patients from the Cancer Hospital, Chinese Academy of Medical Science (CHCAMS cohort), spanning the period from January 2005 to December 2016, and 94 patients from the Peking University Cancer Hospital between January 2010 and April 2023 (PUCH cohort).Archival formalin-fixed paraffin-embedded (FFPE) tumor sections were reviewed by experienced thoracic pathologists. Regions of interest (ROI) were selected and tissue microarrays (TMAs) were constructed. TMA blocks were cut into slides and stained with H&E. All H&E slides were scanned and stored by using the leica Aperio AT2 Scanner (Leica Biosystems) with the x20 objective.
Data analysis	All statistical analyses were performed using R software (version 4.1.3) and relevant R packages. Continuous variables between two groups are compared using a Wilcoxon rank sum test, while categorical variables are compared using Fisher's exact test or the Chi-squared test. Survival curves were generated using the Kaplan-Meier method, and the log-rank test was employed to compare the curves using the R package 'survminer' (version 0.4.9). Cox regression analysis was conducted for univariate and multivariate analyses to estimate the hazard ratios (HR) and corresponding 95% confidence intervals (CI). The source code of this work can be downloaded from https://github.com/ZhoulabCPH/PathoSig

For manuscripts utilizing custom algorithms or software that are central to the research but not yet described in published literature, software must be made available to editors and reviewers. We strongly encourage code deposition in a community repository (e.g. GitHub). See the Nature Portfolio guidelines for submitting code & software for further information.

Data

Policy information about availability of data

All manuscripts must include a data availability statement. This statement should provide the following information, where applicable:

- Accession codes, unique identifiers, or web links for publicly available datasets
- A description of any restrictions on data availability
- For clinical datasets or third party data, please ensure that the statement adheres to our policy

The H&E images and clinical information analyzed during the current study are not publicly available for patient privacy purposes. Data access can be obtained through a reasonable request to Lin Yang (yanglin@cicams.ac.cn). Access to the data will be restricted to non-commercial researches which remove patientsensitive information.

Human research participants

Policy information about studies involving human research participants and Sex and Gender in Research.

Reporting on sex and gender	Among study cohorts, the number of male and female patients is 203 and 83, and 67 and 27 in the CHCAMS and PUCH cohorts, respectively. Male predominance is observed across all cohorts (70% and 76.9% for P-SCLC and C-SCLC in the CHCAMS cohort, and 71.28% for the PUCH cohort).
Population characteristics	The baseline characteristics of the 380 SCLC patients are summarized in Table 1. The PUCH cohort comprised 94 cases of pure SCLC (P-SCLC), while the CHCAMS cohort included 240 P-SCLC cases and 46 combined SCLC (C-SCLC) cases, such as SCLC combined with squamous cell carcinoma (n=19, 41.3%), adenocarcinoma (n=18, 39.1%), large cell carcinoma (n=4, 8.7%), large cell neuroendocrine carcinoma (LCNEC, n=2, 4.3%), carcinoid tumor (n=1, 2.1%), carcinoid tumor and LCNEC (n=1, 2.1%), and adenosquamous carcinoma (n=1, 2.1%). Male predominance is observed across all cohorts (70% and 76.9% for P-SCLC and C-SCLC in the CHCAMS cohort, and 71.28% for the PUCH cohort). The median (range) ages are 56.5 (19-82), 60 (39-76), and 59.5 (33-82) years, and median follow-up durations are 4.00, 4.69, and 3.33 years, and recurrence rates are 49.17%, 50%, and 69.15% for CHCAMS-P-SCLC, CHCAMS-C-SCLC, and PUCH cohorts, respectively. In all cohorts, 141 (58.72%), 24 (52.17%) and 72 (76.60%) cases were in stage I-II, while 99 (41.25%), 22 (47.83%) and 22 (23.40%) cases were in stage III-IV, with lymphatic metastasis observed in 137 (57.08%), 30 (65.22%), and 37 (39.36%) cases across all cohorts.
Recruitment	We retrospectively collected 380 surgically resected and pathologically confirmed specimens of SCLC from two independent medical centers, including 286 patients from the Cancer Hospital, Chinese Academy of Medical Science (CHCAMS cohort), spanning the period from January 2005 to December 2016, and 94 patients from the Peking University Cancer Hospital between January 2010 and April 2023 (PUCH cohort). The inclusion criteria for the study were as follows: (i) Pathologically diagnosed with SCLC, including pure SCLC or combined SCLC; (ii) Availability of complete clinical and pathologic information; (iii) Availability of follow-up data for both disease-free survival (DFS) and overall survival (OS), and (iv) Accessible tumor tissues.
Ethics oversight	This multicenter retrospective study has received ethical approval from the Ethics Committee and Institutional Review Boards of the Cancer Hospital, Chinese Academy of Medical Science (No. 22/250-3452) and Peking University Cancer Hospital (No. 2023KT23). As this was a retrospective study, the requirement for informed consent was waived.

Note that full information on the approval of the study protocol must also be provided in the manuscript.

Field-specific reporting

Please select the one below that is the best fit for your research. If you are not sure, read the appropriate sections before making your selection.

☒ Life sciences ☐ Behavioural & social sciences ☐ Ecological, evolutionary & environmental sciences

For a reference copy of the document with all sections, see nature.com/documents/nr-reporting-summary-flat.pdf

Life sciences study design

All studies must disclose on these points even when the disclosure is negative.

Sample size	Our study contained 286 surgically resected and pathologically confirmed specimens of SCLC from the Cancer Hospital, Chinese Academy of Medical Science (CHCAMS), spanning the period from January 2005 to December 2016, and 94 patients from the Peking University Cancer Hospital between January 2010 and April 2023
Data exclusions	1: Images with blurry, dirty, large areas of blackness and over-stained problems were excluded.<br>2: Absence of thorough clinicopathologicial and follow-up information

Replication	As for each patient, there is no replication. We tested the performance of our model in multi-center cohorts.
Randomization	This design is not relevant to the study; No experimental intervention requiring randomization was applied.
Blinding	Pathologists were blinded to clinical characteristics and prognosis of patients when selecting the regions of interest for features extraction.During the segmentation of all H&E-stained slides, a blind method is employed, which means that the individuals performing the segmentation are unaware of the patients' prognostic information represented by the slides, including DFS (disease-free survival) and OS (overall survival).

Reporting for specific materials, systems and methods

We require information from authors about some types of materials, experimental systems and methods used in many studies. Here, indicate whether each material, system or method listed is relevant to your study. If you are not sure if a list item applies to your research, read the appropriate section before selecting a response.

Materials & experimental systems

n/a	Involved in the study
☒	☐ Antibodies
☒	☐ Eukaryotic cell lines
☒	☐ Palaeontology and archaeology
☒	☐ Animals and other organisms
☒	☐ Clinical data
☒	☐ Dual use research of concern

Methods

n/a	Involved in the study
☒	☐ ChIP-seq
☒	☐ Flow cytometry
☒	☐ MRI-based neuroimaging
